# Stable Walking of a Biped Robot Controlled by Central Pattern Generator Using Multivariate Linear Mapping

**DOI:** 10.3390/biomimetics9100626

**Published:** 2024-10-15

**Authors:** Yao Wu, Biao Tang, Jiawei Tang, Shuo Qiao, Xiaobing Pang, Lei Guo

**Affiliations:** 1 School of Mechatronic Engineering, Changsha University, Changsha 410022, China; tangbiao@mails.guet.edu.cn (B.T.); tangjiawei512@163.com (J.T.); pangxiaobing55@ccsu.edu.cn (X.P.); leiguo@ccsu.edu.cn (L.G.); 2 School of Mechanical and Electrical Engineering, Guilin University of Electronic Technology, Guilin 541004, China

**Keywords:** biped robot, CPG, the limit cycle, multivariate linear mapping

## Abstract

In order to improve the walking stability of a biped robot in multiple scenarios and reduce the complexity of the Central Pattern Generator (CPG) model, a new CPG walking controller based on multivariate linear mapping was proposed. At first, in order to establish a dynamics model, the lower limb mechanical structure of the biped robot was designed. According to the Lagrange and angular momentum conservation method, the hybrid dynamic model of the biped robot was established. The initial value of the robot’s passive walking was found by means of Poincaré mapping and cell mapping methods. Then, a multivariate linear mapping model was established to form a new lightweight CPG model based on a Hopf oscillator. According to the parameter distribution of the new CPG model, a preliminary parameter-tuning idea was proposed. At last, the joint simulation of MATLAB and V-REP shows that the biped robot based on the new CPG control has a stable periodic gait in flat and uphill scenes. The proposed method could improve the stability and versatility of bipedal walking in various environments and can provide general CPG generation and a tuning method reference for robotics scholars.

## 1. Introduction

In the past few decades, robot technology has experienced rapid development, especially the study of humanoid robots, which has made some progress in imitating human behavior and controlling various environmental movements [1,2,3]. As an important branch of robot research, biped robots have received extensive attention due to their potential for flexibility, adaptability, and the ability to walk in complex ground environments. Compared to traditional wheeled or tracked robots, bipedal robots can not only walk on uneven ground but can even climb stairs, which gives them the ability to perform tasks in complex environments, such as disaster relief, home services, and health care [4,5,6].

However, achieving stable and efficient biped walking control has always been one of the great challenges in the field of robotics. This is mainly because biped walking involves complex motion processes and uncertain external environments, which requires the robot to have good balance ability and adaptability. In this context, McGeer [7] proposed a passive dynamic walking biped robot. Unlike traditional biped robots that require continuous external power input, passive biped robots rely on their own gravity and dynamic design to achieve downhill walking without any actuation, thereby significantly reducing energy consumption and simplifying control strategies. However, this pure passive robot has certain limitations and cannot walk effectively in flat or uphill environments. In order to overcome this limitation, researchers have proposed various mathematical algorithms to control biped robots with highly redundant actuators, such as Whole-Body Control (WBC) [8], hybrid zero dynamics (HZD) [9,10], reinforcement learning [11,12,13], demonstration-based imitation learning [14], etc. Compared with the above methods, the biped robot based on bionic control has good adaptability to different environments. This control method does not require accurate and sensitive feedback, nor does it need to establish a complex dynamic model, which greatly reduces the complexity of robot control in a changing environment [15].

As a bionic walking control algorithm, CPG [16,17] is responsible for generating rhythmic motion outputs, such as walking, running, etc., which does not require the participation of advanced neural systems. Many researchers control the biped robot to produce a stable gait by simulating the CPG mechanism, which not only maintains the energy efficiency advantage of the passive biped robot but also significantly improves the robots’ application scope and adaptability [18,19]. Wang et al. used the normalized limit cycle and robot feedback as the feature input, and the neural network model controlled the CPG to generate the required rhythm signal [20]. Pasandi et al. proposed an online adjustment method for the periodic motion of the robot. According to the feedback of the robot motion, an integrated programmable central pattern generator was designed to receive the feedback and generate the reference joint trajectory of the robot system online from the desired periodic motion library [21]. Deshpande et al. proposed a new DeepCPG strategy, which embeds CPG as a layer into a larger neural network and promotes end-to-end learning of motor behavior in deep reinforcement learning (DRL) settings [22]. Akkawutvanich et al. proposed a control method for a planar biped robot based on adaptive parallel reflection and a decoupling center mode generator. The controller adopts a modular structure, which is composed of two parallel modules working together. This method depends on the feedback of the sensor [23]. Bai et al. improved the Hopf oscillator and applied it to CPG control. By adjusting a simple parameter, a hexapod robot can walk in multiple gaits [24]. He et al. proposed a real-time biped walking generation algorithm combining a central pattern generator and foot rotation indication (FRI). According to FRI information, the output of CPG parameters can be adjusted in real time [25]. Liu et al. proposed a hybrid rhythm-reflection control method by combining the CPG network and ZMP stability criterion, which can achieve stable and adaptive biped walking [26]. Yao et al. proposed a hierarchical central pattern generator (H-CPG) model with a basic rhythm signal generation layer and a pattern formation layer to adjust the centroid and online foot trajectory [27]. Liu et al. proposed a new method for designing a CPG model and then used it to control salamander-like robots. The CPG network is composed of an improved Hopf oscillator with a new coupling scheme that can independently control the waveform adjustment and phase coordination process [28]. Wang et al. improved the existing Kimura oscillator model and proposed a new CPG network configuration based on the improved oscillator model for the flat walking gait planning of biped robots [29]. Li et al. proposed a new method called adaptive simulated central pattern generator (AI-CPG), which combines CPG and deep reinforcement learning (DRL) to enhance the motion of humanoid robots [30].

Although robots under CPG controllers could become mobile adaptively by adjusting external feedback parameters or gait behavior library, the success of the robot depends on the delicate design of the CPG walking model, which requires a lot of data support. Furthermore, some of the existing literature directly uses the CPG model for optimization and lacks improvement of the CPG model. The establishment of some improved CPG models is complicated, and the theoretical tuning method of the CPG network parameters has not formed yet.

In order to reduce the complexity of the CPG model and establish a preliminary CPG network parameter-tuning method, this paper proposes a lightweight CPG model to achieve the stable walking of biped robots in various environments. The contribution of this paper is to propose a new lightweight CPG model based on the Hopf oscillator. The model is established by multivariate linear mapping, and the output of the CPG network is adjusted by multiple mapping coefficients to adapt to walking adjustment under different terrains. In the flat and uphill scenarios, the stable walking of the biped robot could be successfully implemented by using the proposed new CPG model. In addition, the distribution law of the new CPG network parameters was analyzed, and a preliminary parameter-tuning idea was proposed. In the structure of the biped robot, this paper proposed a new type of crank rocker mechanism to simplify the establishment of the robot model. The theoretical model of the new CPG is constructed in MATLAB (R2022b), and the control effect of the theoretical model is analyzed. In V-REP (V4.1.0), the new CPG walking controller is applied to the biped robot established in this paper to test whether the biped robot can walk stably. The simulation results of MATLAB and V-REP showed that the CPG based on multivariate linear mapping can control the biped robot to walk stably on flat ground and in uphill environments.

## 2. Mechanical Structure of Biped Robot

**Figure 1:**
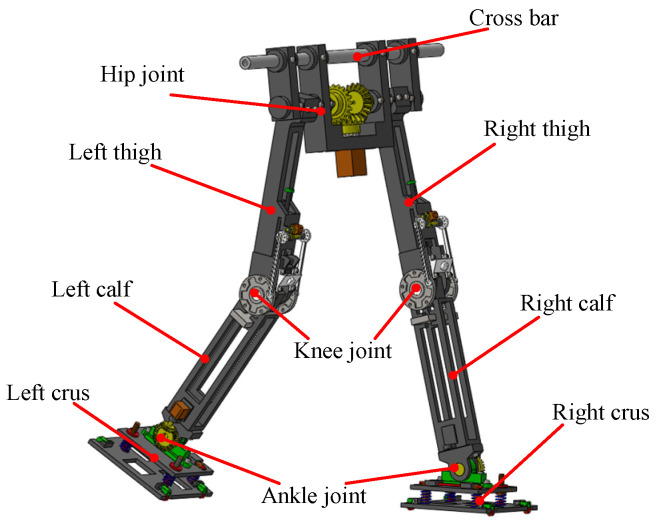
Mechanical structure of biped robot.

In the passive walking control of biped robots, the structural design has a great influence on the initial value search, and it is necessary to reasonably control the distribution of the center of gravity at each joint. The structure design is mainly divided into three parts: the hip joint, knee joint, and ankle joint. The overall structure of the designed robot is symmetrically distributed according to the left and right sides. The height is greater than 0.85 m, and the number of joint degrees of freedom is six. It has the characteristics of light weight and a stable center of gravity. The overall structural design of the robot is shown in Figure 1.

**Figure 2:**
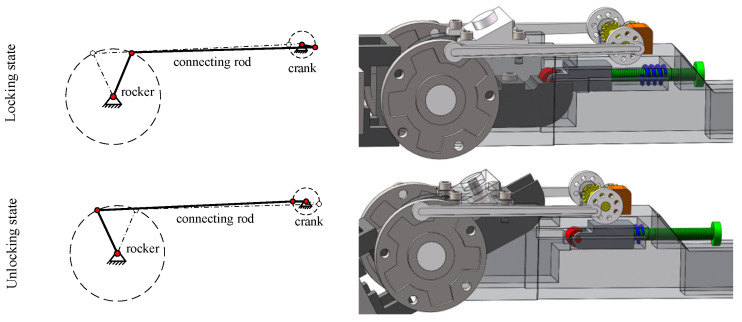
Mechanical structure of knee joint.

The mechanical structure of the hip joint is mainly an angular bisection mechanism, which can effectively stabilize the center of gravity of the hip joint of the robot and improve the walking stability of the robot. The ankle joint has a spring structure to balance the lateral swing force of the biped robot during walking. In this paper, the forward walking stability of the biped robot is studied first, and the analysis of lateral stability will be conducted as a future research topic. In order to further improve walking stability, the mechanical structure of the knee joint of the biped robot is designed. The limit mechanism and locking mechanism are designed at the knee joint, as shown in Figure 2. A new type of crank rocker mechanism is proposed for the limited part of the knee joint. The swing angle of the rocker is consistent with the range of joint swing motion of the calf, which effectively reduces the joint impact. With the gait of the biped robot, the locking mechanism needs to frequently switch the two states of unlocking and locking and has a certain stability. The combination mechanism of roller and spring can reduce the resistance caused by state switching, which is more suitable for the scene of frequent state change.

The design of the mechanical structure of the biped robot can help simplify the dynamic modeling and facilitate the initial value search of passive walking.

## 3. Hybrid Dynamics of Biped Robot

### 3.1. Dynamics of Biped Robot

**Figure 3:**
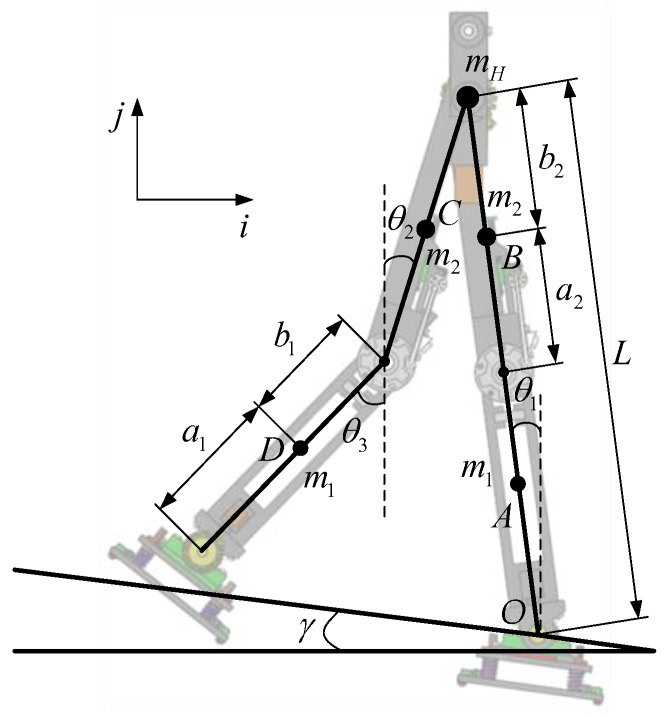
Simplified model of biped robot.

The simplified model of the biped robot walking with knees is established under generalized coordinates, as shown in Figure 3. The model can be simply regarded as a four-bar mechanism, which is symmetrical from both sides of the hip joint. In order to simplify the model, the masses of the hip joint, thigh, and calf are concentrated at their respective centroids, which can be assumed as point mass $m_{H}$, $m_{2}$ and $m_{1}$, respectively. The total length of the leg is L, where the length of the thigh is $l_{2}$ and the length of the calf is $l_{1}$. At the same time, it is necessary to satisfy $l_{2}=a_{2}+b_{2}$, $l_{1}=a_{1}+b_{1}$. The motion of the robot is limited to the vertical plane, and the lateral stability of the biped robot during walking is not considered. The rotational joints between the joints of the biped robot can be driven separately, and the support point and the ground are only one-way constraints, which are non-driven degrees of freedom.

The initial posture of the biped robot is described by the angle. In Figure 3, $\theta_{1}$ is the angle between the supporting leg and the vertical direction. $\theta_{2}$ and $\theta_{3}$ are the angles between the thigh, calf, and the vertical direction of the swing leg, respectively. Because the knee joint of the supporting leg of the biped robot is locked, its posture is expressed by the angle between the supporting leg of the biped robot and the vertical direction. The angle between the inclined plane and the horizontal plane is γ.

The passive walking gait of the biped robot is periodic and is mainly divided into four state phases. Both phase I and phase III are swing phases. The passive walking of the biped robot can be described by the Newton–Lagrange equation and its hybrid dynamic model is established. In phase II and phase IV, the two processes of knee joint collision between the thigh and calf of the swing leg of the biped robot and the foot–ground collision between the swing leg and the ground can be described by the angular momentum conservation theorem. The function relationship between the state of the biped robot system before and after the collision can be given by the algebraic mapping method.

The following assumptions were made for the walking process of the biped robot in order to simplify its dynamics:

1. The support foot has maintained contact with the ground, and there is no relative slip whether the biped robot is in the single-legged support phase or double-legged support phase.
2. In the support leg support stage, the knee joint collision between the thigh and calf of the swing leg begins, and the collision process is instantaneous.
3. The contact process between the swing leg and the ground is assumed to be a completely elastic collision, and the collision time is instantaneous.

In phase I, the knee joint locking mechanism of the support leg remains locked, and the swing leg bends naturally. The Lagrangian function of the oscillating phase is as follows:

(1) $$M(\theta )\ddot{\theta }+C(\theta ,\dot{\theta })\dot{\theta }+g(\theta )=\mathrm{Bu}$$

where *θ* is the joint angle vector of the robot, and *Bu* represents the external torque of each joint of the robot. M(θ) is the inertia matrix of the biped robot, $C(\theta ,\dot{\theta })$ is the Coriolis matrix of the dynamic equation, and g(θ) is the gravity vector of the biped robot. For the passive walking biped robot, $u={\left[0,\;0,\;0\right]}^{T}$.

Let *x* =$\;{\left[\theta ,\;\dot{\theta }\right]}^{T},\;f\left(x\right)=\dot{x}$, and rewrite Equation (1) into the form of state space, as follows:

(2) $$f(x)=\dot{x}=\left[\begin{array}{c} \dot{\theta }\\ \ddot{\theta } \end{array}\right]=\left[\begin{array}{c} \dot{\theta }\\ M^{-1}(\theta )[-C(\theta ,\dot{\theta })\dot{\theta }-g(\theta )+Bu] \end{array}\right]$$

where $f\left(x\right)$ is the equation of the state of the oscillating phase.

In phase II, the swing leg moves to the straight state. At this time, the thigh and the calf are in the same straight line and the knee joint begins to lock and remains locked in the subsequent state until the next gait cycle begins. The dynamic equations of the biped robot before and after knee joint collision can be obtained by using the principle of angular momentum conservation. Its state space is expressed as follows:

(3) $$x^{+}=\Delta_{1}(x^{-}),\Delta_{1}=\left[\begin{array}{c} \begin{array}{ccc} 1 & 0 & 0 \end{array}\\ \begin{array}{ccc} 0 & 1 & 0 \end{array}\\ {\left(Q_{1}^{+}\right)}^{-1}Q_{1}^{-} \end{array}\right]$$

where $x^{-}={{[\theta }_{1},\theta_{2},\;\theta_{3},\dot{\theta_{1}},\;\dot{\theta_{2}},\;\dot{\theta_{3}}]}^{T}$ is the state variable of the robot before collision, and $x^{+}=[{\theta_{1},\;\theta_{2},\;\dot{\theta_{1}},\;\dot{\theta_{2}}]}^{T}$ is the state variable after collision. After the collision is completed, the state variables change from six to four.

The robot dynamics equation of phase III is similar to that of phase I. The dynamic equation of phase IV is similar to that of phase II. The details of dynamics equations are provided in Appendix A.

### 3.2. Initial Value Solution of Passive Walking

The pure passive walking of the biped robot on the slope is completely dependent on the gravity and inertia of the robot itself. The gait equation of the robot is a complex multi-mode hybrid system, and numerical simulation is usually used to obtain its stable passive walking solution. According to the mechanical structure of the biped robot in Figure 1, its physical parameters can be obtained, as shown in Table 1. The data in Table 1 are measured by the mechanical model of the biped robot in the SolidWorks software (2018). The software provides related functions to help analyze the established mechanical model. For the numerical simulation of the passive walking of biped robots, those physical parameters will be used.

**Figure 4:**
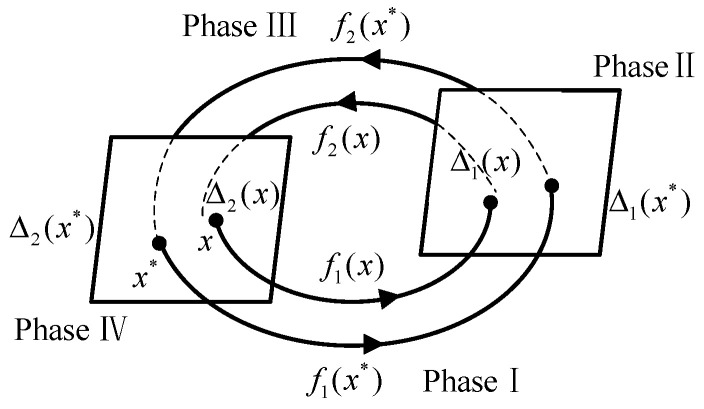
Poincaré section.

The passive walking of the biped robot is mainly affected by the mechanical structure of the robot and the initial state of the robot. The initial state of the robot mainly includes the angle and angular velocity of each structure of the robot under generalized coordinates. Based on the gait cycle of the passive walking of biped robots with knees, the Poincaré section can select the knee joint collision surface (phase II in Figure 4), the foot–ground collision surface (phase IV in Figure 4), etc. In general, the foot–ground collision state surface is selected as the Poincaré section. At this time, the passive gait parameter dimension of the robot is reduced from six to four, which greatly simplifies the calculation amount, and the result will be closer to the fixed point of the periodic gait (x* in Figure 4).

**Figure 5:**
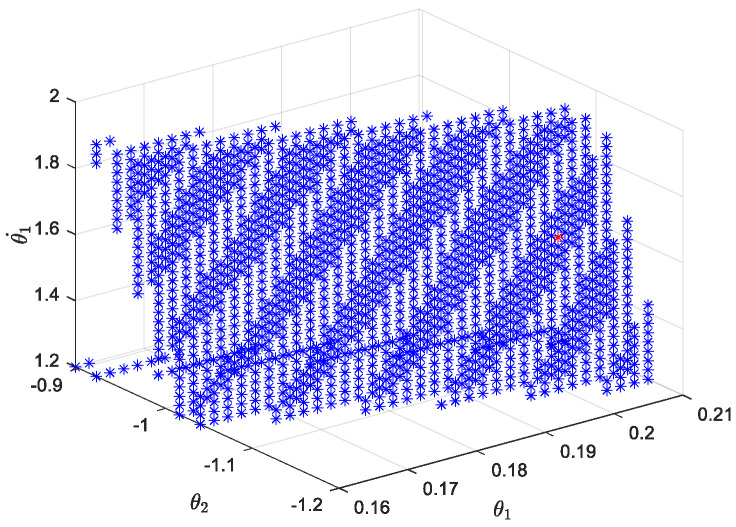
Attractive region of passive gait periodic solution for biped robot.

After the Poincaré section is selected, the periodic domain of attraction is solved using the cell mapping method [31]. The selected ground tilt angle is 0.1001 rad; the discrete state space is $\theta_{1}\;$∈ [0.16, 0.21], $\theta_{2}$ ∈ [−1.4, −0.8], $\dot{\theta_{1}}$ ∈ [1.2, 1.9], $\dot{\theta_{2}}$ ∈ [1.1, 1.8]; and the number of cells is 160,000. The periodic attraction domain of passive walking calculated by MATLAB (R2022b) is shown in Figure 5.

Due to the limited number of discrete space cells, the calculation of the cell mapping method has certain accuracy limitations. However, in the domain of attraction solved by the cell mapping method, the periodic solution close to the fixed point x* can be obtained as [$\theta_{1},\;\theta_{2},\;\dot{\theta_{1}},\;\dot{\theta_{2}}]\;$= [0.206, −1.088, 1.564, 1.6040]. The red point in Figure 5 is the periodic solution close to the fixed point found by the mathematical model of cell mapping.

In order to find the fixed point of the periodic motion of the semi-passive biped robot, the mathematical model of the Poincaré mapping method [32] is established in MATLAB. Substituting the periodic solution near the fixed point obtained by the cell mapping method, the initial value $x_{0}$ to generate periodic solution of the robot’s passive walking is as follows:

(4) $$x_{0}=[\theta_{1},\theta_{2},\theta_{3},{\dot{\theta }}_{1},{\dot{\theta }}_{2},{\dot{\theta }}_{3}]=[0.1925,-0.3919,-0.3921,-1.0746,1.5121,1.3789]$$

## 4. Walking Control of Biped Robot Based on New CPG

### 4.1. CPG Controller Based on Multivariate Linear Mapping

The Hopf oscillator has the merit of generating a stable limit cycle, which could be applied to the legged robot to plan stable motion. In this paper, a control strategy of the biped robot based on a Hopf oscillator is proposed. The oscillator model is established as follows:

(5) $$\left\{\begin{array}{l} \dot{x}=\alpha \left(u-x^{2}-y^{2}\right)x-\omega y\\ \dot{y}=\beta \left(u-x^{2}-y^{2}\right)y+\omega x \end{array}\right.$$

where *x* and *y* are two state variables of nonlinear differential equations, which are self-excited oscillation functions of time. *α* and *β* can control the convergence rate of the oscillator; $\sqrt{u}$ is the amplitude of the oscillator; ω is the vibration frequency of the oscillator.

In order to control the joint torque of the biped robot more accurately, a multivariate linear mapping model is established based on a Hopf oscillator. The model can control the output of the basic oscillator by adjusting the parameters. In different terrains, the biped robot can achieve stable walking by adjusting the parameters of the model. The CPG model based on multivariate linear mapping is as follows:

(6) $$\left\{\begin{array}{l} \dot{x}=\alpha \left(u-x^{2}-y^{2}\right)x-\omega y\\ \dot{y}=\beta \left(u-x^{2}-y^{2}\right)y+\omega x\\ X=X_{a}+X_{R}x\\ Y=Y_{a}+Y_{R}y \end{array}\right.$$

where *x* and *y* are Hopf oscillator output results by Equation (5); $X_{a}$ and $Y_{a}$ are the offsets of the self-excited oscillation function relative to the initial position; $X_{R}$ and $Y_{R}$ are the amplitudes of the self-oscillating function curves; *X* and *Y* are the outputs of the CPG model based on multivariate linear mapping.

Compared with other CPG establishment methods for improving Hopf oscillators [28,33], the method proposed in this paper is a lightweight model. The overall complexity of the model is low, and there is no need to introduce more mathematical methods. On the basis of the core Hopf oscillator, the establishment of multivariate linear mapping can be directly applied to the joint torque of the biped robot. The multivariate linear mapping method proposed in this paper can reduce the number of Hopf oscillators, and it is not necessary to set an independent Hopf oscillator at each joint. Only one Hopf oscillator is set in each swing phase, and the output of the oscillator is adjusted by a finite number of coefficients at each joint, so as to control the motion of each joint.

**Figure 6:**
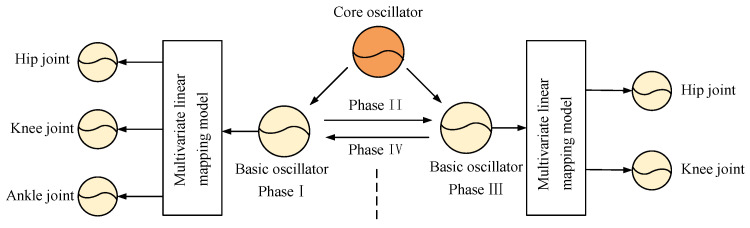
CPG network topology based on multivariate linear mapping model.

The biped robot with knees contains two swing phases in a periodic gait (phase I, phase III). Therefore, it is necessary to use the CPG model to control the joint torque of the two swing phases of the biped robot. The topology of the CPG controller based on the multivariate linear mapping model is shown in Figure 6. According to the characteristics of biped robot walking, a basic oscillator is established in two swing phases, respectively. This basic oscillator is universal and does not need to be adjusted after its establishment. In different terrain scenes, only adjusting the parameters of the multivariate linear mapping model to change the control torque of each joint can achieve stable walking of the biped robot.

In order to verify the effectiveness of the new CPG controller, a theoretical model is constructed in MATLAB (R2022b), and the walking control analysis of the biped robot in flat and uphill scenarios is given, respectively.

### 4.2. Walk on Flat Ground

For phase I, the multivariate linear mapping model of the new CPG model in the flat scene is:

(7) $$\left\{\begin{array}{l} X_{1}=X_{a1}+X_{R1}x_{1}\\ Y_{1}=Y_{a1}+Y_{R1}y_{1}\\ Z_{1}=Z_{a1}+Z_{R1}y_{1} \end{array}\right.$$

where $x_{1}$ and $y_{1}$ are the self-excited oscillation functions obtained after setting the relevant parameters in Equation (6). In the Lagrange dynamic equation in phase I, $u={\left[X_{1},Y_{1},Z_{1}\right]}^{T}$ represents the torque control applied to the hip joint, knee joint, and ankle joint.

The parameter settings of each part are as follows:

(8) $$\left\{\begin{array}{l} \alpha =1,\;\beta =1,\;u=3,\;\omega =7\pi ,\;x_{1}(0)=2,\;y_{1}(0)=0;\\ X_{a1}=6.5,\;X_{R1}=4.5;\;\;\;Y_{a1}=0.4,\;Y_{R1}=0.9;\\ Z_{a1}=0.46,\;Z_{R1}=0.2 \end{array}\right.$$

**Figure 7:**
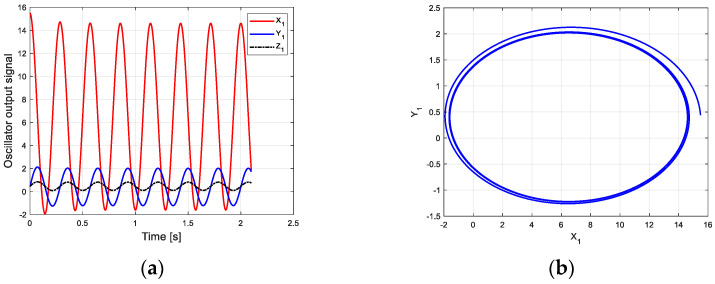
Oscillation curve of each joint in phase I on flat ground. (**a**) Oscillator output curve; (**b**) phase space trajectory.

According to this parameter and the new CPG model, the oscillation curve and phase space trajectory of each joint in phase I can be obtained in MATLAB, as shown in Figure 7. Figure 7a is the oscillation signal of the control torque of each joint. Figure 7b shows the joint torque oscillation space formed by $X_{1}$ and $Y_{1}$. The closed limit cycle formed by the oscillation space indicates that the output signal is relatively stable.

For phase III, the multivariate linear mapping model of the new CPG model in the flat scene is as follows:

(9) $$\left\{\begin{array}{l} X_{2}=X_{a2}+X_{R2}x_{2}\\ Y_{2}=Y_{a2}+Y_{R2}y_{2} \end{array}\right.$$

where $x_{2}$ and $y_{2}$ are the self-excited oscillation functions obtained after setting the relevant parameters in Equation (6). In the Lagrange dynamic equation in phase I, $u={\left[X_{2},Y_{2}\right]}^{T}$ represents the torque control applied to the hip joint and knee joint.

The parameter settings of each part are as follows:

(10) $$\left\{\begin{array}{l} \alpha =1,\;\beta =1,\;u=3,\;\omega =8\pi ,\;x_{2}(0)=3,\;y_{2}(0)=0;\\ X_{a2}=-85,\;X_{R2}=35;\;\;\;Y_{a2}=-2.6,\;Y_{R2}=2; \end{array}\right.$$

**Figure 8:**
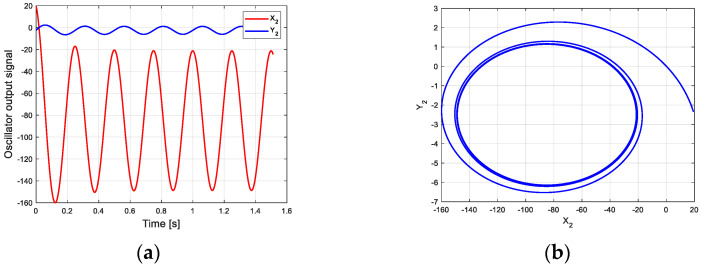
Oscillation curve of each joint in phase III on flat ground. (**a**) Oscillator output curve; (**b**) phase space trajectory.

According to this parameter and the new CPG model, the oscillation curve and phase space trajectory of each joint in phase III can be obtained in MATLAB, as shown in Figure 8. Figure 8a is the oscillation signal of the control torque of each joint. Figure 8b shows the joint torque oscillation space formed by $X_{2}$ and $Y_{2}$. The closed limit cycle formed by the oscillation space indicates that the output signal is relatively stable. Compared with the limit cycle of oscillation space in Figure 7b, the initial position of Figure 8b has a large deviation.

**Figure 9:**
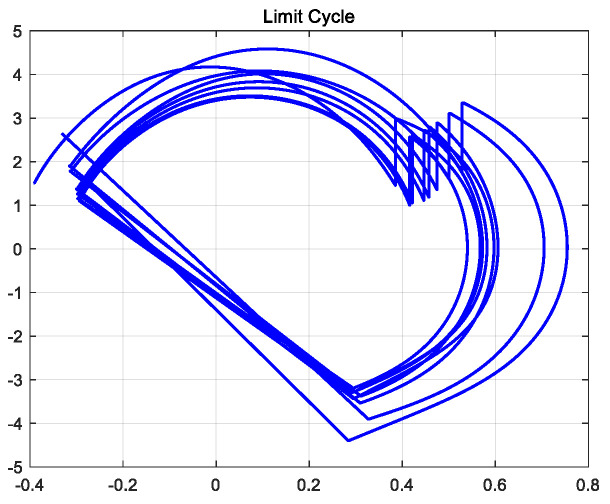
Robot walking limit cycle on flat ground.

Under the action of the controller, the biped robot can walk stably on flat ground. In the hybrid dynamic model of the biped robot established by MATLAB, the limit cycle of its stable walking can be obtained, as shown in Figure 9. The initial conditions of biped robot walking are consistent with the initial conditions of robot passive walking. The CPG model based on multivariate linear mapping is used to control the joint torque of the robot. After two steps of walking, the robot began to gradually adapt to the transition from the slope to the flat ground. The walking adjustment was stable and the anti-interference ability was strong.

**Figure 10:**
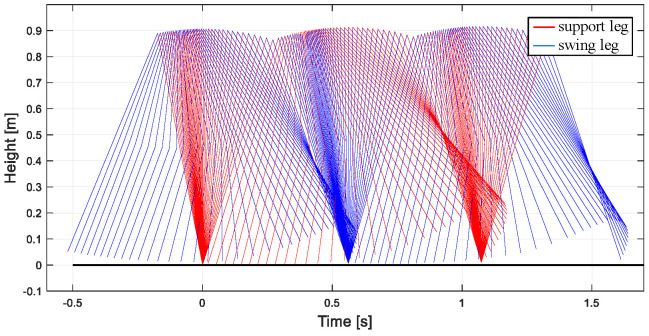
Robot walking posture on flat ground based on new CPG.

The gait simulation stick figure of the robot can be obtained in MATLAB, as shown in Figure 10. The simulation results show that the walking gait of the biped robot is similar to the passive walking gait under the inclined plane, and the control method can make the biped robot walk stably.

**Figure 11:**
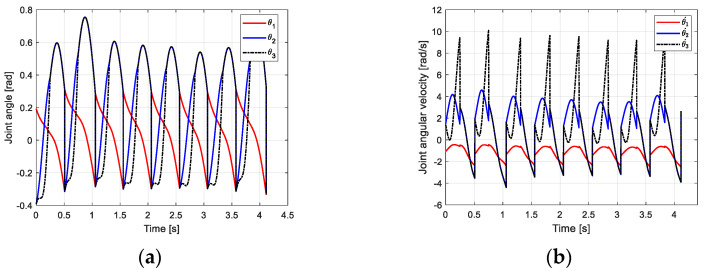
Motion relationship of robot walking on flat ground. (**a**) Joint velocity curve; (**b**) angular velocity curve of joint.

From the joint angle change curve of the robot walking on the flat ground obtained in Figure 11a, under the influence of the CPG controller, the robot gradually adjusts from the slope to the flat ground through two steps, and the transition is gentle and stable. The change of joint angular velocity is shown in Figure 11b. The change law is consistent with the passive gait law of the robot, indicating that the CPG control method based on multivariate linear mapping can effectively realize the stable gait walking of the biped robot in multiple scenarios and improve the adaptability of the robot to the environment.

### 4.3. Walking on the Uphill

For phase I, the multivariate linear mapping model of the new CPG model in the uphill scene is:

(11) $$\left\{\begin{array}{l} X_{11}=X_{a11}+X_{R11}x_{11}\\ Y_{11}=Y_{a11}+Y_{R11}y_{11}\\ Z_{11}=Z_{a11}+Z_{R11}y_{11} \end{array}\right.$$

where $x_{11}$ and $y_{11}$ are the self-excited oscillation functions obtained after setting the relevant parameters in Equation (6). In the Lagrange dynamic equation in phase I, $u={\left[X_{11},Y_{11},Z_{11}\right]}^{T}$ represents the torque control applied to the hip joint, knee joint, and ankle joint.

The parameter settings of each part are as follows:

(12) $$\left\{\begin{array}{l} \alpha =1,\;\beta =1,\;u=3,\;\omega =7\pi ,\;x_{11}(0)=2.1,\;y_{11}(0)=0;\\ X_{a11}=14,\;X_{R11}=3.57;\;\;\;Y_{a11}=2.31,\;Y_{R11}=0.4;\\ Z_{a11}=0.7,\;Z_{R11}=0.4 \end{array}\right.$$

**Figure 12:**
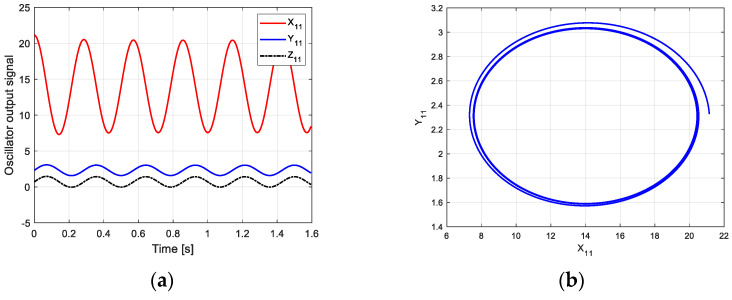
Oscillation curve of each joint in phase I on the uphill. (**a**) Oscillator output curve; (**b**) phase space trajectory.

According to this parameter and the new CPG model, the oscillation curve and phase space trajectory of each joint in phase I can be obtained in MATLAB, as shown in Figure 12. Figure 12a is the oscillation signal of the control torque of each joint. The oscillation amplitudes of $Y_{11}$ and $Z_{11}$ are almost the same. Figure 12b shows the joint torque oscillation space formed by $X_{11}$ and $Y_{11}$. The closed limit cycle formed by the oscillation space indicates that the output signal is relatively stable.

For phase III, the multivariate linear mapping model of the new CPG model in the uphill scene is as follows:

(13) $$\left\{\begin{array}{l} X_{22}=X_{a22}+X_{R22}x_{22}\\ Y_{22}=Y_{a22}+Y_{R22}y_{22} \end{array}\right.$$

where $x_{22}$ and $y_{22}$ are the self-excited oscillation functions obtained after setting the relevant parameters in Equation (6). In the Lagrange dynamic equation in phase I, $u={\left[X_{22},Y_{22}\right]}^{T}$ represents the torque control applied to the hip joint and knee joint.

The parameter settings of each part are as follows:

(14) $$\left\{\begin{array}{l} \alpha =1,\;\beta =1,\;u=3,\;\omega =8\pi ,\;x_{22}(0)=3,\;y_{22}(0)=0;\\ X_{a22}=-124,\;X_{R22}=35;\;\;Y_{a22}=-15,\;Y_{R22}=6; \end{array}\right.$$

**Figure 13:**
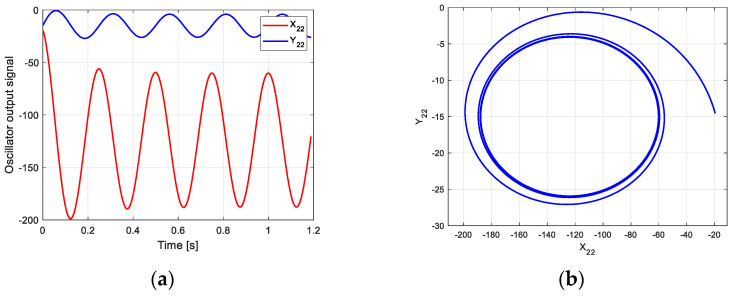
Oscillation curve of each joint in phase III on the uphill. (**a**) Oscillator output curve; (**b**) phase space trajectory.

According to this parameter and the new CPG model, the oscillation curve and phase space trajectory of each joint in phase III can be obtained in MATLAB, as shown in Figure 13. Figure 13a is the oscillation signal of the control torque of each joint. Figure 13b shows the joint torque oscillation space formed by $X_{22}$ and $Y_{22}$. The closed limit cycle formed by the oscillation space indicates that the output signal is relatively stable.

**Figure 14:**
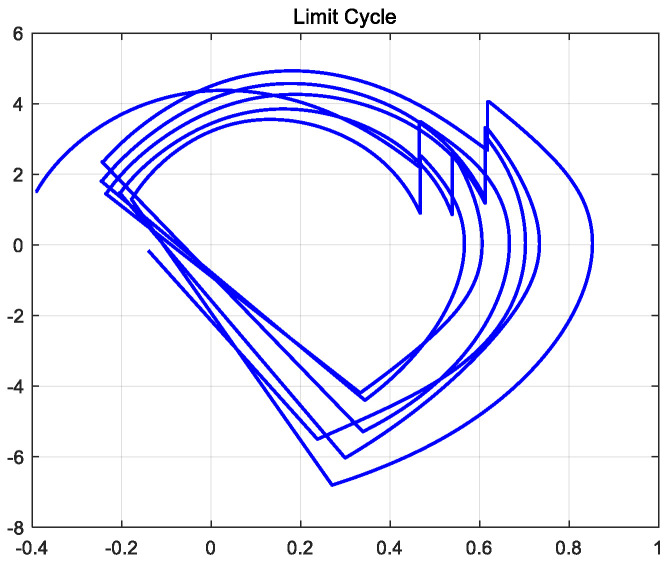
Robot walking uphill limit cycle.

Under the action of the controller, the semi-passive biped robot can walk stably uphill (that is, the tilt angle of the ground is 0.05 rad). In the hybrid dynamic model of the biped robot established by MATLAB, the limit cycle of its stable walking can be obtained, as shown in Figure 14. The initial condition of the biped robot walking is consistent with the initial condition of the robot passive walking. The Hopf oscillator is used to control the joint torque of the robot. After a few steps of walking, the robot gradually adapts to the transition from the downhill slope to the uphill slope, and the walking adjustment is more stable.

**Figure 15:**
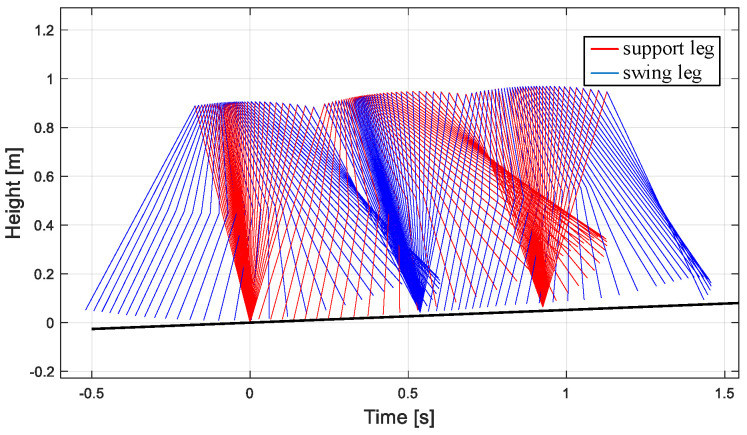
The uphill walking posture of the robot based on the new CPG.

The gait simulation stick figure of the robot can be obtained in MATLAB, as shown in Figure 15. The simulation results show that the walking gait of the biped robot is similar to the passive walking gait under the inclined plane, and the control method can make the biped robot walk stably.

**Figure 16:**
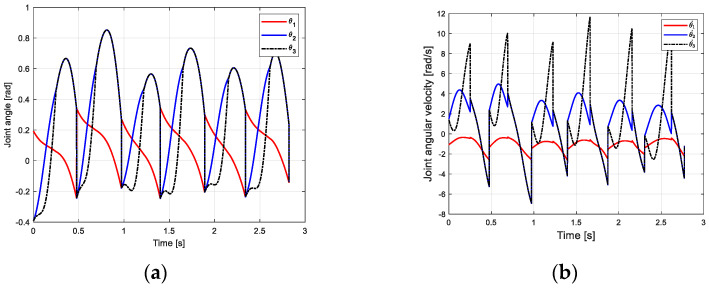
The motion curve of the robot walking uphill. (**a**) Joint velocity curve; (**b**) angular velocity curve of joint.

From the joint angle change curve of the robot walking on the flat ground obtained in Figure 16a, under the influence of the CPG controller, the robot gradually adjusts from the downhill slope to the uphill slope through three steps, and the transition has certain fluctuations. Finally, the area is stable under the control of the CPG. The time required for CPG to adjust to a stable state in different scenarios is different. This is because the initial value of the robot is obtained under the downhill condition. The closer the slope is to the slope, the fewer steps are required, and vice versa. The change in joint angular velocity is shown in Figure 16b. The change law is consistent with the passive gait law of the robot, indicating that the CPG control method based on multivariate linear mapping can effectively realize the stable gait walking of the biped robot in multiple scenarios and improve the adaptability of the robot to the environment.

### 4.4. General CPG Parameter-Tuning Idea

**Figure 17:**
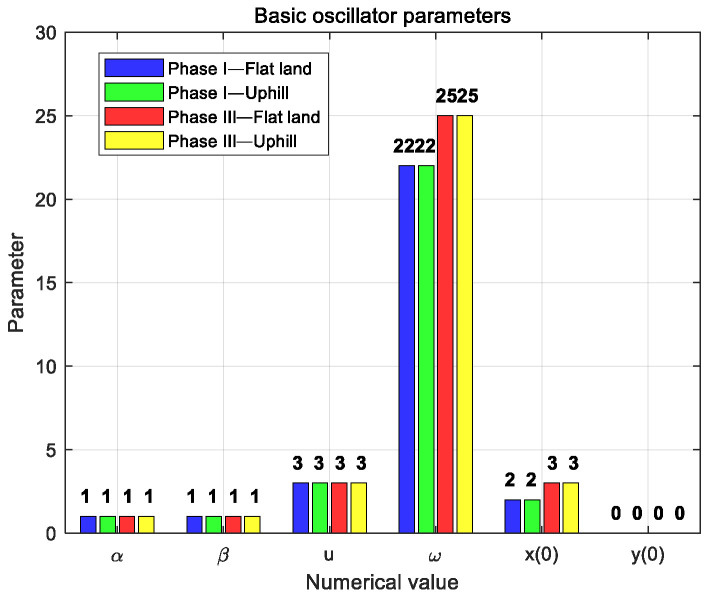
Comparison of related parameters of basic oscillator.

According to the CPG parameters obtained in the two scenarios of flat ground and uphill, it can be found that the parameters of the basic oscillator are basically the same in the gait phase I (i.e., Equations (8) and (12)) and phase III (i.e., Equations (10) and (14)), that is, parameter $\left[\alpha ,\beta ,u,\omega ,x\left(0\right),y\left(0\right)\right]$. These parameters have a certain universality and do not need to adjust the parameters according to the changes in the walking scene, so they can be initialized first. Comparing the gait phase I and phase III, the basic oscillator needs to adjust the frequency parameter ω and the starting position x(0). The comparison of the related parameters of the basic oscillator under different gaits and terrains is shown in Figure 17.

**Figure 18:**
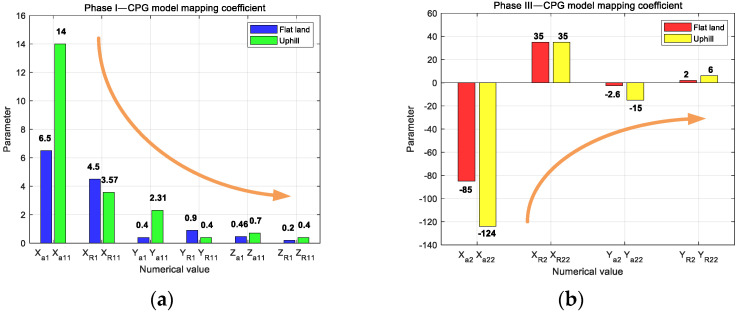
Comparison of mapping coefficients of CPG model. (**a**) Phase I; (**b**) phase III.

The multivariate linear mapping coefficients in the CPG model are shown in Figure 18. The mapping coefficient [X,Y,Z] corresponds to the hip joint, knee joint, and ankle joint of the biped robot, respectively. In Figure 18a phase I, the value of parameter $\left[X_{a},\;X_{R}\right]$ is larger, indicating that the torque applied at the hip joint is larger. The value of parameter $\left[Z_{a},\;Z_{R}\right]$ is small, indicating that the torque applied at the ankle joint is small. The arrows in the figure indicate that from the hip joint, knee joint, to ankle joint, the relevant parameter values show a downward trend. In different terrains, most of the parameters in phase I are close and positive. When changing from flat ground to uphill conditions, parameter $\left[X_{a},Y_{a},\;Z_{a}\right]$ shows an increasing trend.

In phase III of Figure 18b, the absolute value of parameter $\left[X_{a},\;X_{R}\right]$ is large, indicating that the torque applied at the hip joint is large. The absolute value of parameter $\left[Y_{a},\;Y_{R}\right]$ is small, indicating that the torque applied at the knee joint is small. The arrows in the figure indicate that from the hip joint to the knee joint, the relevant parameter values show an upward trend. The absolute value of the parameters also meets the requirements of joint torque control. In different terrains, most of the parameters in phase III are close, but the values are positive and negative. When changing from flat to uphill conditions, parameter $\left[X_{a},Y_{a}\right]$ shows an increasing trend in absolute value. The absolute value of the parameters in phase III is about eight times that of the absolute value of the parameters in phase I, and the gap between some parameters is larger or smaller.

The preliminary general tuning idea of the CPG controller parameters based on the multivariate linear mapping model is as follows:

1. Firstly, according to the gait phase I and III, the basic oscillator model is established, respectively, and the oscillator parameter $\left[\alpha ,\beta ,u,\omega ,x\left(0\right),y\left(0\right)\right]$ is initialized.
2. Establish a multivariate linear mapping model that matches phase I and III.
3. According to the parameter distribution of phase I and phase III, the CPG parameters of the stable walking of the biped robot on terrain are preliminarily adjusted. The parameters in phase I are preset, and the mapping parameters are assigned according to the trend from large to small. In phase III, the parameters are assigned according to eight times the absolute value of the parameters in phase I, and the sign changes are noted. According to the obtained biped robot walking limit cycle, the CPG parameters are fine-tuned.
4. The optimization algorithm [34] is used to adjust the CPG parameters of the biped robot in other terrains near a set of parameters successfully debugged.

The general CPG parameter-tuning idea proposed in this paper is helpful in establishing the CPG parameter-tuning theory of the system and promotes the further improvement of the CPG walking control theory of the biped robot.

## 5. Walking Results under CPG

**Figure 19:**
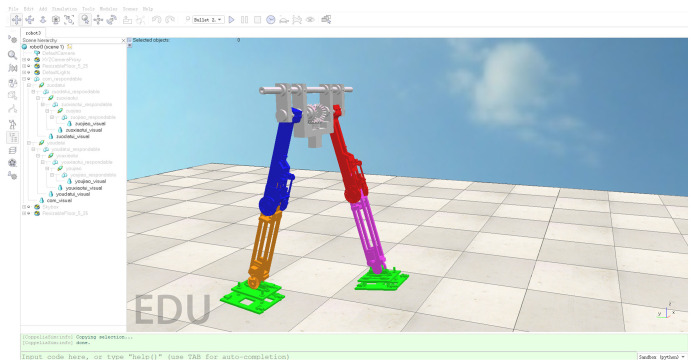
Semi-passive biped robot in V-REP.

In order to verify the feasibility of the CPG controller based on multivariate linear mapping, the scene will be built in V-REP (V4.1.0) and the motion simulation of the robot will be carried out. Firstly, the mechanical structure model established in SolidWorks is imported into V-REP (as shown in Figure 19), the connection relationship of the relative rotation of the hip joint, knee joint, and ankle joint is determined, and the simulation model tree of biped robot is established. In order to enable the semi-passive biped robot to be statically balanced, a flat scene is built in the V-REP, the initial joint angles of the support leg and the swing leg are set according to the CPG control method, and the maximum driving torque of each joint is set.

**Figure 20:**
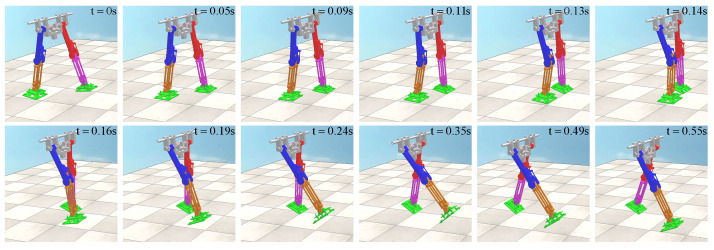
Robot walking in flat ground scene in V-REP.

Then, the joint simulation channel of V-REP and MATLAB is established, and the control program based on CPG is used to drive the biped robot model in V-REP in MATLAB. In the flat ground scene of V-REP, the single-step walking gait of the biped robot is shown in Figure 20. For flat ground walking, the biped robot does not fall and can achieve stable walking, indicating that the method proposed in this paper has certain feasibility. The biped robot uses about 0.55 s for one-step walking, which is consistent with the analysis in MATLAB. At 0.24 s, the biped robot collided with the knee joint, and the locking mechanism locked the robot’s thigh and calf together. In the subsequent motion, the robot’s thigh and calf maintain a consistent motion relationship until the motion relationship changes after the foot–ground collision.

**Figure 21:**
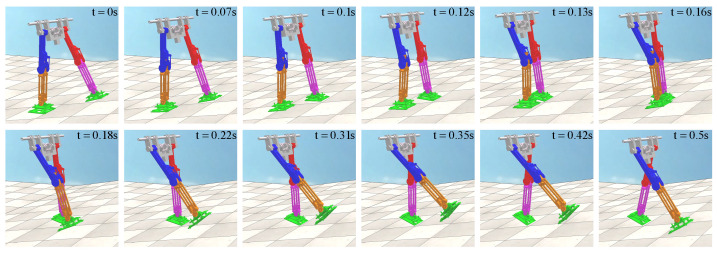
Robot walking in uphill scene in V-REP.

In the uphill scene of the V-REP, the tilt angle of the ground is 0.05 rad, and the one-step walking gait of the biped robot is shown in Figure 21. For uphill walking, the biped robot did not fall and could achieve stable walking, which again verified the feasibility of the method in this paper. The biped robot uses about 0.5 s for one-step walking, which is faster than the walking speed of the flat ground scene. At 0.22 s, the biped robot collided with the knee joint, and the locking mechanism locked the robot’s thigh and calf together. In order to maintain dynamic stability, the biped robot will reduce the walking time compared with the flat ground scene when it goes uphill.

In the simulation of V-REP, the connection of each joint is simplified. The simulation results can verify the feasibility of the CPG control method based on multivariate linear mapping and realize the stable walking of the biped robot in multiple scenarios.

## 6. Conclusions and Future Work

The biped robot structure model established in this paper can facilitate the initial value search of passive walking. A limit and locking mechanism was designed at the knee joint of the robot, which can provide support for the stable walking of the biped robot. According to the mechanical structure of the robot, a hybrid dynamic model is established, and the pure passive motion gait of the robot under the slope is analyzed. The initial conditions of robot walking are found by the Poincaré and cell mapping methods.

In order to establish a new lightweight CPG controller, a multivariate linear mapping model is proposed based on the Hopf oscillator, and a new CPG model is established. After simulation by MATLAB and V-REP, the results show that the CPG based on multivariate linear mapping can control the biped robot to walk stably in multiple scenes, such as flat ground and uphill. In addition, the distribution law of the new CPG network parameters is analyzed, and a preliminary parameter-tuning idea is proposed. The relevant parameters of the mapping model provide a reference case for the robot based on this method, which is convenient to further promote research on the control method of environmental adaptability of the biped robot. The CPG establishment method based on multivariate linear mapping proposed in this paper is a lightweight model. Compared with algorithms involving artificial intelligence, the method in this paper does not require a lot of training data support, and the model complexity is low. The method in this paper has good performance on biped robots, and the control of other multi-legged robots needs to be further studied.

In the future, the parameters of the lightweight new CPG model will be optimized by the proposed parameter-tuning idea and the optimization algorithm or machine learning algorithm. In addition, a prototype of the biped robot will be established and experiments will be carried out to verify the proposed method.

## Figures and Tables

**Table 1:** Mechanical structure parameters of biped robot.

Parameter Name	Sign	Values [Unit]
Leg length	$L=l_{1}\;$ $+l_{2}$	0.9062 [m]
Waist mass	$m_{H}$	11 [kg]
Leg mass	$m_{1}$	2.2 [kg]
Thigh mass	$m_{2}$	11 [kg]
Centroid position of calf	$a_{1}$ $/l_{1}$	0.614
Centroid position of thigh	$a_{2}$ $/l_{2}$	0.468
Acceleration of gravity	*g*	9.8 [m s^−2^]

## Data Availability

The data that support the findings of this study are available upon request from the corresponding author. The data are not publicly available due to privacy and ethical restrictions.

## Appendix A

### Appendix A.1. Passive Walking Gait Modeling of Underactuated Biped Robot

Both phase I and phase III are swing phases. The passive walking of the biped robot can be described by the Newton–Lagrange equation, and its hybrid dynamic model is established. In phase II and phase IV, the two processes of knee joint collision and foot–ground collision between the swing leg of the biped robot and the ground can be described by the angular momentum conservation theorem.

#### Appendix A.1.1. Phase I

The kinetic and potential energy expressions of the robot are as follows:

(A1) $$K\left(\theta ,\dot{\theta }\right)=\frac{1}{2}m_{H}{\left({\vec{v}}_{H}\right)}^{2}+\frac{1}{2}m_{1}{\left({\vec{v}}_{11}\right)}^{2}+\frac{1}{2}m_{2}{\left({\vec{v}}_{12}\right)}^{2}+\frac{1}{2}m_{1}{\left({\vec{v}}_{3}\right)}^{2}+\frac{1}{2}m_{2}{\left({\vec{v}}_{2}\right)}^{2}$$

(A2) $$P\left(\theta \right)=\left(m_{H}+m_{2}+m_{2}\right)gL\cos\theta_{1}+m_{1}ga_{1}\cos\theta_{1}+m_{2}g\left(l_{1}+a_{1}\right)\cos\theta_{1}-\left(b_{2}m_{2}+m_{1}l_{2}\right)g\cos\theta_{2}-m_{1}gb_{1}\cos\theta_{3}$$

In the generalized coordinates $\left[\vec{i,}\;\vec{j}\right]$, the detailed expression of the speed of each part of the robot is:

(A3) $$\begin{array}{l} {\vec{v}}_{H}=-L{\dot{\theta }}_{1}\cos\theta_{1}\vec{i}-L{\dot{\theta }}_{1}sin\theta_{1}\vec{j}\\ {\vec{v}}_{11}=-a_{1}{\dot{\theta }}_{1}\cos\theta_{1}\vec{i}-a_{1}{\dot{\theta }}_{1}sin\theta_{1}\vec{j}\\ {\vec{v}}_{12}=-\left(l_{1}+a_{2}\right){\dot{\theta }}_{1}\cos\theta_{1}\vec{i}-\left(l_{1}+a_{2}\right){\dot{\theta }}_{1}sin\theta_{1}\vec{j}\\ {\vec{v}}_{2}=\left(-L{\dot{\theta }}_{1}\cos\theta_{1}+b_{2}{\dot{\theta }}_{2}\cos\theta_{2}\right)\vec{i}+\left(-L{\dot{\theta }}_{1}\sin\theta_{1}+b_{2}{\dot{\theta }}_{2}\sin\theta_{2}\right)\vec{j}\\ {\vec{v}}_{3}=\left(-L{\dot{\theta }}_{1}\cos\theta_{1}+b_{1}{\dot{\theta }}_{3}\cos\theta_{3}+l_{2}{\dot{\theta }}_{2}\cos\theta_{2}\right)\vec{i}+\left(-L{\dot{\theta }}_{1}\sin\theta_{1}+b_{1}{\dot{\theta }}_{3}\sin\theta_{3}+l_{2}{\dot{\theta }}_{2}\sin\theta_{2}\right)\vec{j} \end{array}$$

The specific expression of the robot’s kinetic energy is:

(A4) $$\begin{array}{ll} K\left(\theta ,\dot{\theta }\right)= & \frac{1}{2}\left[m_{1}a_{1}^{2}+m_{2}{\left(l_{1}+a_{2}\right)}^{2}+\left(m_{H}+m_{1}+m_{2}\right)L^{2}\right]{{\dot{\theta }}_{1}}^{2}+\frac{1}{2}\left(m_{2}b_{2}^{2}+m_{1}l_{2}^{2}\right){{\dot{\theta }}_{2}}^{2}+\frac{1}{2}m_{2}b_{1}^{2}{{\dot{\theta }}_{3}}^{2}\\ & -\left(m_{2}b_{2}+m_{1}l_{2}\right)L\cos\left({\dot{\theta }}_{2}-\theta_{1}\right){\dot{\theta }}_{1}{\dot{\theta }}_{2}-m_{1}b_{1}L\cos\left(\theta_{3}-\theta_{1}\right){\dot{\theta }}_{1}{\dot{\theta }}_{3}+m_{1}b_{1}l_{1}\cos\left(\theta_{3}-\theta_{2}\right){\dot{\theta }}_{2}{\dot{\theta }}_{3} \end{array}$$

According to the Newton–Lagrange function:

(A5) $$\frac{d}{dt}\left(\frac{\partial L\left(\theta ,\dot{\theta }\right)}{\partial \dot{\theta }}\right)-\frac{\partial L\left(\theta ,\dot{\theta }\right)}{\partial \theta }=0$$

The dynamic equation of the robot is:

(A6) $$M_{1}\left(\theta \right)\ddot{\theta }+C_{1}\left(\theta ,\dot{\theta }\right)\dot{\theta }+g_{1}\left(\theta \right)=B_{1}u$$

Among them, matrix $M=\left(\begin{array}{ccc} m_{11} & m_{12} & m_{13}\\ m_{21} & m_{22} & m_{23}\\ m_{31} & m_{32} & m_{33} \end{array}\right)$ is a symmetric matrix, and the specific expression of each element in the matrix is:

(A7) $$\begin{array}{l} m_{11}=m_{1}a_{1}^{2}+m_{2}{\left(l_{1}+a_{2}\right)}^{2}+\left(m_{H}+m_{1}+m_{2}\right)L^{2},\;m_{21}=m_{12},\;m_{31}=m_{13}\\ m_{12}=-\left(m_{2}b_{2}L+m_{1}Ll_{2}\right)\cos\left(\theta_{2}-\theta_{1}\right),\;m_{22}=m_{2}b_{2}^{2}+m_{1}l_{2}^{2},\;m_{32}=m_{23}\\ m_{13}=-m_{1}Lb_{1}\cos\left(\theta_{3}-\theta_{1}\right),\;m_{23}=m_{1}l_{2}b_{1}\cos\left(\theta_{3}-\theta_{2}\right),\;m_{33}=m_{1}b_{1}^{2} \end{array}$$

Matrix $C=\left(\begin{array}{ccc} c_{11} & c_{12} & c_{13}\\ c_{21} & c_{22} & c_{23}\\ c_{31} & c_{32} & c_{33} \end{array}\right)$ is an anti-symmetric matrix, and the specific expression of each element is:

(A8) $$\begin{array}{l} c_{11}=0,\;\;\;\;\;\;\;\;\;\;\;\;c_{12}=\left(m_{2}b_{2}L+m_{1}l_{2}L\right){\dot{\theta }}_{2}\sin\left(\theta_{2}-\theta_{1}\right),\\ c_{21}=-\left(m_{2}b_{2}L+m_{1}l_{2}L\right){\dot{\theta }}_{1}\sin\left(\theta_{2}-\theta_{1}\right),\;c_{22}=0,\\ c_{31}=m_{1}b_{2}L{\dot{\theta }}_{1}\sin\left(\theta_{1}-\theta_{3}\right),\;\;\;\;\;c_{32}=m_{1}b_{1}l_{2}{\dot{\theta }}_{2}\sin\left(\theta_{3}-\theta_{2}\right),\\ c_{13}=m_{1}b_{1}L{\dot{\theta }}_{3}\sin\left(\theta_{3}-\theta_{1}\right),\;\;\;\;\;c_{23}=-m_{1}b_{1}l_{2}{\dot{\theta }}_{3}\sin\left(\theta_{3}-\theta_{2}\right),c_{33}=0 \end{array}$$

In matrix $g_{1}={\left[g_{1}g_{2}g_{3}\right]}^{T}$, the specific expression of each element is:

(A9) $$\begin{array}{l} g_{1}=-\left(m_{H}+m_{1}+m_{2}\right)gL\sin\theta_{1}-m_{1}ga_{1}\sin\theta_{1}-m_{2}g\left(l_{1}+a_{2}\right)\sin\theta_{1},\\ g_{2}=\left(m_{1}l_{2}+m_{2}b_{2}\right)g\sin\theta_{2},\;\;\;\;\;g_{3}=m_{1}gb_{1}\sin\theta_{3} \end{array}$$

If each joint can exert a driving torque, then:

(A10) $$B_{1}=\left(\begin{array}{ccc} 1 & -1 & 0\\ 0 & 1 & 0\\ 0 & 0 & 1 \end{array}\right),u={\left[u_{1}u_{2}u_{3}\right]}^{T}$$

#### Appendix A.1.2. Phase II

In the knee joint collision, the whole re-support leg of the robot is subjected to external force, and the angular momentum of the whole support point is conserved. The swing leg is only affected by the external force at the hip joint, and the angular momentum of the swing leg about the hip joint is conserved. The formula is as follows:

(A11) $$\begin{array}{l} m_{1}\bar{OA}\times {\bar{V}}_{A}^{-}+m_{2}\bar{OB}\times {\bar{V}}_{B}^{-}+m_{H}\bar{OH}\times {\bar{V}}_{H}^{-}+m_{2}\bar{OC}\times {\bar{V}}_{C}^{-}+m_{1}\bar{OD}\times {\bar{V}}_{D}^{-}\\ =m_{1}\bar{OA}\times {\bar{V}}_{A}^{+}+m_{2}\bar{OB}\times {\bar{V}}_{B}^{+}+m_{H}\bar{OH}\times {\bar{V}}_{H}^{+}+m_{2}\bar{OC}\times {\bar{V}}_{C}^{+}+m_{1}\bar{OD}\times {\bar{V}}_{D}^{+} \end{array}$$

(A12) $$m_{2}\bar{HC}\times {\bar{V}}_{C}^{-}+m_{1}\bar{HD}\times {\bar{V}}_{D}^{-}=m_{2}\bar{HC}\times {\bar{V}}_{C}^{+}+m_{1}\bar{HD}\times {\bar{V}}_{D}^{+}$$

Before and after the knee joint collision, the angular momentum of the robot is conserved.

(A13) $$Q_{1}^{+}{\left[{\dot{\theta }}_{1}^{+}{\dot{\theta }}_{2}^{+}\right]}^{T}=Q_{1}^{-}{\left[{\dot{\theta }}_{1}^{-}{\dot{\theta }}_{2}^{-}{\dot{\theta }}_{3}^{-}\right]}^{T}$$

Among them, in matrix $Q_{1}^{-}=\left[\begin{array}{l} \begin{array}{ccc} Q_{11}^{-} & Q_{12}^{-} & Q_{13}^{-} \end{array}\\ \begin{array}{ccc} Q_{21}^{-} & Q_{22}^{-} & Q_{23}^{-} \end{array} \end{array}\right]$, the specific expression of each element is:

(A14) $$\begin{array}{l} Q_{11}^{-}=-\left(m_{1}l_{2}+m_{2}b_{2}\right)L\cos\left(a\right)-m_{1}b_{1}L\cos\left(b\right)+\left(m_{1}+m_{2}+m_{H}\right)L^{2}+m_{1}a_{1}^{2}+m_{2}{\left(l_{1}+a_{2}\right)}^{2},\\ Q_{12}^{-}=-\left(m_{1}l_{2}+m_{2}b_{2}\right)L\cos\left(a\right)+m_{1}b_{1}L\cos\left(c\right)+m_{2}b_{2}^{2}+m_{1}l_{2}^{2},\\ Q_{13}^{-}=-m_{1}b_{1}L\cos\left(b\right)+m_{1}b_{1}l_{2}\cos\left(c\right)+m_{1}b_{1}^{2},\;\;Q_{21}^{-}=-\left(m_{1}l_{2}+m_{2}b_{2}\right)L\cos\left(a\right)-m_{1}b_{1}L\cos\left(b\right),\\ Q_{22}^{-}=m_{1}b_{1}l_{2}\cos\left(c\right)+m_{2}b_{2}^{2}+m_{1}l_{2}^{2},\;\;\;\;\;\;Q_{23}^{-}=m_{1}b_{1}l_{2}\cos\left(c\right)+m_{1}b_{1}^{2} \end{array}$$

In matrix $Q_{1}^{+}=\left[\begin{array}{cc} \begin{array}{l} Q_{11}^{+}\\ Q_{21}^{+} \end{array} & \begin{array}{l} Q_{12}^{+}\\ Q_{22}^{+} \end{array} \end{array}\right]$, the specific expression of each element is:

(A15) $$\begin{array}{l} Q_{11}^{+}=Q_{21}^{+}+m_{2}{\left(l_{1}+a_{2}\right)}^{2}+\left(m_{H}+m_{1}+m_{2}\right)L^{2}+m_{1}a_{1}^{2},Q_{12}^{+}=Q_{21}^{+}+m_{1}{\left(l_{2}+b_{1}\right)}^{2}+m_{2}b_{2}^{2},\\ Q_{21}^{+}=-\left[m_{1}\left(l_{2}+b_{1}\right)+m_{2}b_{2}\right]L\cos\left(a\right),\;\;\;\;\;\;Q_{22}^{+}=m_{1}{\left(l_{2}+b_{1}\right)}^{2}+m_{2}b_{2}^{2}, \end{array}$$

Among them, $a=\theta_{1}-\theta_{2},\;b=\theta_{1}-\theta_{3},\;c=\theta_{2}-\theta_{3}$.

#### Appendix A.1.3. Phase III

The swing phase is similar to phase I, and the dynamic model of the robot in phase III can be obtained directly.

The dynamic equation of the robot is:

(A16) $$M_{2}\left(\theta \right)\ddot{\theta }+C_{2}\left(\theta ,\dot{\theta }\right)\dot{\theta }+g_{2}\left(\theta \right)=B_{2}u$$

Among them, matrix $M_{2}=\left[\begin{array}{cc} \begin{array}{l} m_{11}\\ m_{21} \end{array} & \begin{array}{l} m_{12}\\ m_{22} \end{array} \end{array}\right]$ is a symmetric matrix, and the specific expression of each element in the matrix is:

(A17) $$\begin{array}{l} m_{11}=m_{1}a_{1}^{2}+m_{2}{\left(l_{1}+a_{2}\right)}^{2}+\left(m_{H}+m_{1}+m_{2}\right)L^{2},\;m_{21}=m_{12},\\ m_{12}=-\left[m_{2}b_{2}L+m_{1}L\left(l_{2}+b_{1}\right)\right]\cos\left(\theta_{2}-\theta_{1}\right),\;m_{22}=m_{2}b_{2}^{2}+m_{1}{\left(l_{2}+b_{1}\right)}^{2} \end{array}$$

Matrix $C_{2}=\left[\begin{array}{cc} \begin{array}{l} c_{11}\\ c_{21} \end{array} & \begin{array}{l} c_{12}\\ c_{22} \end{array} \end{array}\right]$ is an anti-symmetric matrix, and the specific expression of each element is:

(A18) $$\begin{array}{l} c_{11}=0,\;\;c_{12}=\left[-m_{2}b_{2}L-m_{1}L\left(l_{2}+b_{1}\right)\right]{\dot{\theta }}_{2}\sin\left(\theta_{1}-\theta_{2}\right),\\ c_{21}=\left[m_{2}b_{2}L+m_{1}L\left(l_{2}+b_{1}\right)\right]{\dot{\theta }}_{1}\sin\left(\theta_{1}-\theta_{2}\right),c_{22}=0, \end{array}$$

In matrix $g_{1}={\left[g_{1}g_{2}\right]}^{T}$, the specific expression of each element is:

(A19) $$\begin{array}{l} g_{1}=-\left(m_{H}+m_{1}+m_{2}\right)gL\sin\theta_{1}-m_{1}ga_{1}\sin\theta_{1}-m_{2}g\left(l_{1}+a_{2}\right)\sin\theta_{1},\\ g_{2}=\left[m_{2}b_{2}+m_{1}\left(l_{2}+b_{1}\right)\right]g\sin\theta_{2} \end{array}$$

If each joint can exert a driving torque, then:

(A20) $$B_{2}=\left[\begin{array}{cc} \begin{array}{l} 1\\ 0 \end{array} & \begin{array}{l} -1\\ 1 \end{array} \end{array}\right],u={\left[u_{1}u_{2}\right]}^{T}$$

#### Appendix A.1.4. Phase IV

In the foot collision stage, the robot swing leg foot is affected by the ground reaction force (set the collision point as *O*). The angular momentum conservation of the robot as a whole is at the impact of the swing leg foot. Since the supporting leg before the collision is only subjected to the force at the hip joint, the angular momentum of the supporting leg about the hip joint is conserved.

(A21) $$\begin{array}{l} m_{1}\bar{OA}\times {\bar{V}}_{A}^{-}+m_{2}\bar{OB}\times {\bar{V}}_{B}^{-}+m_{H}\bar{OH}\times {\bar{V}}_{H}^{-}+m_{2}\bar{OC}\times {\bar{V}}_{C}^{-}+m_{1}\bar{OD}\times {\bar{V}}_{D}^{-}\\ =m_{1}\bar{OA}\times {\bar{V}}_{A}^{+}+m_{2}\bar{OB}\times {\bar{V}}_{B}^{+}+m_{H}\bar{OH}\times {\bar{V}}_{H}^{+}+m_{2}\bar{OC}\times {\bar{V}}_{C}^{+}+m_{1}\bar{OD}\times {\bar{V}}_{D}^{+} \end{array}$$

(A22) $$m_{2}\bar{HA}\times {\bar{V}}_{A}^{-}+m_{1}\bar{HB}\times {\bar{V}}_{B}^{-}=m_{2}\bar{HA}\times V_{A}^{+}+m_{1}\bar{HB}\times {\bar{V}}_{B}^{+}$$

Before and after the foot–ground collision, the angular momentum of the robot at the collision point and the angular momentum of the supporting leg at the hip joint are conserved.

(A23) $$Q_{2}^{+}{\left[{\dot{\theta }}_{1}^{+}{\dot{\theta }}_{2}^{+}\right]}^{T}=Q_{2}^{-}{\left[{\dot{\theta }}_{1}^{-}{\dot{\theta }}_{2}^{-}\right]}^{T}$$

Among them, in matrix $Q_{1}^{-}=\left[\begin{array}{cc} \begin{array}{l} Q_{11}^{-}\\ Q_{21}^{-} \end{array} & \begin{array}{l} Q_{12}^{-}\\ Q_{22}^{-} \end{array} \end{array}\right]$, the specific expression of each element is:

(A24) $$\begin{array}{l} Q_{11}^{-}=Q_{12}^{-}+\left[m_{H}L+2m_{2}\left(l_{1}+a_{2}\right)+m_{1}a_{1}\right]L\cos\left(a\right),\;Q_{21}^{-}=Q_{22}^{-},\\ Q_{12}^{-}=-m_{1}a_{1}\left(l_{2}+b_{1}\right)-m_{2}b_{2}\left(l_{1}+a_{2}\right),\;\;\;\;\;\;Q_{22}^{-}=0 \end{array}$$

In matrix $Q_{2}^{+}=\left[\begin{array}{cc} \begin{array}{l} Q_{11}^{+}\\ Q_{21}^{+} \end{array} & \begin{array}{l} Q_{12}^{+}\\ Q_{22}^{+} \end{array} \end{array}\right]$, the specific expression of each element is:

(A25) $$\begin{array}{l} Q_{11}^{+}=Q_{21}^{+}+m_{2}{\left(l_{1}+a_{2}\right)}^{2}+\left(m_{H}+m_{1}+m_{2}\right)L^{2}+m_{1}a_{1}^{2},\;Q_{12}^{+}=Q_{21}^{+}+m_{1}{\left(l_{2}+b_{1}\right)}^{2}+m_{2}b_{2}^{2},\\ Q_{21}^{+}=-\left[m_{1}\left(l_{2}+b_{1}\right)+m_{2}b_{2}\right]L\cos\left(a\right),\;\;\;\;\;\;\;Q_{22}^{+}=m_{1}{\left(l_{2}+b_{1}\right)}^{2}+m_{2}b_{2}^{2}, \end{array}$$

Among them, $a=\theta_{1}-\theta_{2},\;b=\theta_{1}-\theta_{3},\;c=\theta_{2}-\theta_{3}$.
